# Editorial: Case reports in intensive care medicine 2023

**DOI:** 10.3389/fmed.2025.1563533

**Published:** 2025-02-18

**Authors:** Cheng Zhu, Chen Chen, Bin Lin, Yuetian Yu

**Affiliations:** ^1^Department of Disease Prevention and Control, Ruijin Hospital, Shanghai Jiao Tong University, School of Medicine, Shanghai, China; ^2^Department of Critical Care Medicine, Renji Hospital, School of Medicine, Shanghai Jiao Tong University, Shanghai, China; ^3^Key Laboratory of Intelligent Pharmacy and Individualized Therapy of Huzhou, Huzhou, China; ^4^Department of Pharmacy, Changxing People's Hospital, Changxing Branch, Second Affiliated Hospital of Zhejiang University School of Medicine, Huzhou, China

**Keywords:** case report, intensive care unit, critical care medicine, sepsis, acute respiratory distress syndrome, precision medicine

Critical care medicine has undergone nearly 70 years of development and has now become a relatively mature discipline primarily focused on the treatment of organ failure syndromes (1). The future direction of the discipline is toward translational medicine and precision medicine, which requires further clarification of the pathogenesis of heterogeneous syndromes such as sepsis or acute respiratory distress syndrome (ARDS) from the perspective of sub-phenotypes to achieve precise treatment (2, 3). At the same time, it encourages the organization and summarization of medical histories for rare and uncommon critical illnesses, as well as the publication of related reports to provide experience for the precise treatment of relevant diseases in the future (4, 5).

Therefore, we have compiled this Research Topic on “*Case Reports in Intensive Care Medicine*,” aiming for an in-depth discussion in the relevant fields. We are also pleased to receive a lot of submissions, and some of them have been successfully published after revisions. This Research Topic mainly focuses on the following areas.

## Heterogeneity among critically ill patients

Critically ill patients exhibit significant heterogeneity in clinical characteristics, etiology, clinical response, and prognosis, which has important implications for treatment planning and clinical management (1, 6, 7). Specifically, the heterogeneity of critically ill patients manifests in several key areas: First, the etiology of critically ill patients may include infections (such as sepsis), trauma, postoperative complications, cardiovascular diseases, and respiratory failure, with different etiologies leading to varied pathophysiological changes and treatment needs. However, even patients with similar diagnoses may present vastly different clinical manifestations. For example, patients with sepsis may exhibit varying degrees of fever, hypotension, and altered consciousness, primarily influenced by factors such as the patient's baseline health status, age, sex, and comorbidities, all of which affect their response to critical illness. Second, there is currently a requirement for stratified individual immune status assessments for critically ill patients. Patients show significant differences in their immune system status, some may exhibit excessive inflammatory responses while others may be immunosuppressed. These variations in immune response can affect the severity of infections and treatment outcomes. Additionally, immune status impacts the heterogeneity of the host's response to treatment, leading to significant differences in how different patients respond to the same treatment regimen. Some patients may improve rapidly, while others may show no obvious response or experience adverse reactions. Third, the ability to metabolize drugs varies among patients due to individual differences, influencing both the efficacy and safety of medications, which ultimately may impact patient prognosis, resulting in some patients recovering well from critical conditions while others face a higher risk of mortality or long-term sequelae (8).

Due to this heterogeneity, the management of critically ill patients requires individualized treatment strategies tailored to each patient's specific circumstances, thereby enhancing treatment efficacy and improving prognosis.

## Requirements for individualized precision treatment in critically ill patients

Critically ill patients often present with complex conditions, typically characterized by multiple causes leading to similar outcomes. Therefore, beyond adhering to broad treatment protocols (such as the bundled approach for sepsis), there is an urgent need for individualized precision treatment to improve patient prognosis (9). This includes the following points:

1. Accurate diagnosis of etiology: critically ill patients usually have complex conditions, and quickly and accurately identifying the source of infection or other causes is fundamental to developing an individualized treatment plan. This requires advanced diagnostic technologies, such as molecular biology tests and imaging examinations.
2. Individualized drug selection: based on the specific condition of the patient, characteristics of the pathogens, and their drug resistance, the most appropriate antibiotics or other therapeutic agents should be selected. This includes considering factors such as the patient's age, gender, underlying diseases, and allergy history.
3. Adjustment of drug dosage: the metabolism of drugs in critically ill patients may be influenced by various factors, such as liver and kidney function and hemodynamic status. Therefore, individualized adjustment of drug dosage is crucial to ensure therapeutic efficacy and reduce adverse reactions.
4. Comprehensive treatment plans: critically ill patients often require collaboration among multidisciplinary teams, including specialists in infectious diseases, critical care medicine, respiratory medicine, and nephrology, to formulate comprehensive treatment plans that cover anti-infective therapy, fluid resuscitation, nutritional support, and mechanical ventilation.
5. Real-time monitoring and assessment: the condition of critically ill patients can change rapidly, necessitating dynamic monitoring to adjust treatment plans promptly. This includes monitoring vital signs, biochemical indicators, and infection markers.
6. Management of drug resistance: with the increasing prevalence of drug-resistant bacteria, special attention must be paid to resistance issues in the treatment of critically ill patients, adjusting antibiotic usage strategies in a timely manner to avoid unnecessary use of broad-spectrum antibiotics.
7. Physiological and psychological support for patients: in addition to physiological treatment, critically ill patients also require psychological support and humane care to help them cope with the anxiety and fear associated with their critical condition.
8. Family and social support: the understanding and involvement of critically ill patients and their families in the treatment plan are also important components of individualized treatment. Providing relevant information and support helps patients and families make informed decisions.
9. Prognosis evaluation and long-term management: after discharge, critically ill patients still require attention to their long-term health status. Therefore, individualized prognosis evaluation and follow-up management are also very important for the timely identification and management of potential issues.
10. Accumulating evidence from evidence-based medicine to form guidelines: there is significant heterogeneity among critically ill patients, and currently, there are few treatment guidelines specifically targeting certain sub-phenotypes or genotypes of diseases. Therefore, case reports are needed to transition to case series studies, further accumulating treatment experiences.

## Case reports play a significant role in the field of critical care medicine

The causes of illness in critically ill patients are complex, and the related pathophysiological changes occur rapidly, leading to considerable heterogeneity. To pursue the goal of individualized precision treatment, case reports are essential in critical care medicine for the following reasons:

1. Clinical experience sharing: case reports can detail and share the diagnosis, treatment processes, and outcomes of specific patients. This provides valuable references for other clinicians, especially when dealing with rare or complex cases, allowing them to draw on others' experiences to reduce misdiagnosis and treatment delays.
2. Promoting knowledge dissemination: through case reports, healthcare professionals can learn about the latest clinical practices and research findings. This helps improve the overall standards in critical care medicine and promotes the application of new technologies and methods.
3. Identifying new diseases and variants: case reports can assist in recognizing newly emerging diseases, variant strains, or rare complications. In critical care medicine, timely detection of these changes is crucial for improving diagnosis and treatment plans as well as formulating preventive measures.
4. Facilitating early diagnosis: by reviewing case reports, doctors can understand the manifestations and progression of different types of sepsis, thereby increasing awareness of early symptoms and facilitating quicker diagnoses and interventions to reduce mortality rates.
5. Exploring new treatment strategies: case reports help summarize and analyze the effectiveness of various treatment methods in sepsis, including antibiotic selection, fluid resuscitation, and supportive therapies. Sharing successful cases can provide new ideas and approaches for clinical practice.
6. Identifying rare complications: sepsis may be accompanied by various complications, and case reports can help clinicians recognize these rare but significant complications, enhancing the accuracy of clinical judgment and guiding subsequent treatment strategies.
7. Enhancing education and training: case reports are an important component of medical education. They provide real clinical cases for medical students and residents, serving as a foundation for continuing education and helping healthcare professionals continually update their knowledge.
8. Promoting research development: in-depth analysis of cases allows researchers to propose new hypotheses and research questions, advancing both basic and clinical research. This contributes to exploring disease mechanisms, optimizing treatment plans, and improving patient outcomes.
9. Strengthening multidisciplinary collaboration: the management of sepsis often requires the collaboration of multidisciplinary teams. Case reports can demonstrate the roles and contributions of different disciplines in the management of sepsis, fostering teamwork and improving overall treatment efficacy.
10. Enhancing patient safety: by summarizing and analyzing cases, potential risk factors and adverse events can be identified, providing suggestions for improving clinical practice, ultimately enhancing patient safety and treatment outcomes.

## Summary of the Research Topic and future directions

In summary, case reports in critical care medicine are not only a reflection of clinical practice but also an important form of academic communication. They play an indispensable role in promoting the dissemination of medical knowledge, enhancing clinical skills, and advancing scientific research. We encourage the submission and publication of case reports and hope to receive further support from everyone in the future compilation of related content, as well as more high-quality case report manuscripts.
